# HNRNPA2B1 is a potential biomarker of breast cancer related to prognosis and immune infiltration

**DOI:** 10.18632/aging.204992

**Published:** 2023-09-05

**Authors:** Aisikeer Ayoufu, Lina Yi, Muhairemu Tuersuntuoheti, Yongtao Li

**Affiliations:** 1 Department of Breast Surgery, Xinjiang Medical University Affiliated Tumor Hospital, Urumqi, Xinjiang 830011, China

**Keywords:** m6A, HNRNPA2B1, prognosis, immune infiltration, breast cancer

## Abstract

Objective: HNRNPA2B1, one of the regulator of m6A methylation, is involved in a wide range of physiological processes. However, the aberrant expression of HNRNPA2B1 in Breast Cancer (BC) and its clinical significance still need to be further studied.

Methods: We used related databases to analyze the relationship between HNRNPA2B1 and BC by bioinformatics. Then, we further detected the expression of HNRNPA2B1 by immunohistochemical method, and analyzed the relationship between it and the prognosis of breast cancer by COX regression method.

Results: In the study, we found that the expression level of HNRNPA2B1 in breast cancer (BC) was significantly higher than that in normal breast tissues. In addition, the expression level of HNRNPA2B1 in BC samples was significantly correlated with clinical indexes such as TNM stage. The Cox analysis revealed that the expression of HNRNPA2B1 in BC had significant clinical prognostic value. The results of immune infiltration of HNRNPA2B1 showed that there was a significant correlation between HNRNPA2B1 and immune cell subsets.

Conclusion: Our results show that the expression of HNRNPA2B1 in BC has important clinical diagnostic significance and high expression may be related with poor clinical outcome of BC. This helps to provide us with a new direction of BC targeted therapy.

## INTRODUCTION

Recently, the incidence of breast cancer has been increasing minimally. According to the latest World Health Organization report, the prevalence of this cancer accounts for the highest proportions among malignancies, and it also the most prevalent women cancers (24.5%) [1]. The etiology of breast cancer was complicated with genetic and environmental events [2]. And the genetic background usually determines therapeutic effect, prognosis of patients, and even the tumorigenesis [3].

Our previous studies demonstrated that BRCA1/2 [4] and PALB2 [5] mutation increases the risk of breast cancer. The post-transcriptional modification involved in a plethora aspects of physiological processes, such as RNA processing and metabolism, and was also complicated in cancer development [6]. Methylation modification accounts for more than 2/3 of various types of RNA modification, and these modifications exist widely in various RNA types [7, 8]. N6-methyladenosine (m6A), which happens at the N6 position of adenosine, is the most common internal modification of eukaryotic RNA [9]. HNRNPA2B1 is one of the important members of m6A.

HNRNPA2B1, the RNA nuclear binding protein, mainly involved in RNA splicing, mRNA processing modification, synthesis of telomeres, repair of DNA damage, regulation of gene expression and protein translation and other complex biological processes [10]. It is suggested that driven cancer initiation through interaction with other proteins [11]. It also leads to tumor progression by regulating the expression of malignant tumor genes, improving the proliferation and migration of cancer cells, and inhibiting tumor cell apoptosis [12].

In this study, we utilized multi-dimensional investigation to explore the underlying oncogenic mechanism of HNRNPA2B1 in breast cancer, including bioinformatical analysis and human tissues examination. The results of this study presented that HNRNPA2B1 is a potential diagnostic and prognostic marker of breast cancer.

## MATERIALS AND METHODS

### Data source

Use GDC API (https://portal.gdc.cancer.gov/) to download TCGA-BRCA’s RNAseq data in level 3 HTSeq-FPKM format on April 20, 2021, including 1099 cancer samples and 114 normal samples. Exclusion criteria: (1) there is no complete RNA sequencing data in the sample; (2) samples treatment naive; (3) advanced breast cancer. Finally, 1092 cases of breast cancer and 113 cases of normal breast tissue were included Supplementary Table 1.

### Selection and differential expression analysis of m6A methylation regulators

According to the related literature [8, 11, 13], we selected 28 m6A methylation regulators that regulate RNA methylation, including 10 writers, and 2 erasers, 11 readers. The differential expression analysis of 28 m6A regulators Supplementary Table 2 in breast cancers and normal controls was compared by the “limma” package in R, heatmap was assessed by the “Complex Heatmap” package, and violin map was drawn by the “ggplot2” package.

### Construction and validation of diagnostic score model by m6A methylation regulator

Lasso regression analysis was used to screen the most valuable factors from the candidate m6A regulators. All samples from TCGA were randomly classified into training group and test group. Lasso regression analysis was performed with training group to construct the diagnostic score (DS) model, DS = exp gene (1) × β1 + exp gene (2) × β2 + … exp gene (*n*) × βn. The diagnostic signature of the model was validated by receiver operating characteristic (ROC) curve in test group and GEO datasets. Meanwhile, the tumor samples were divided into high-score group and low-score group by median diagnostic score, to indicated the distinguish between high score groups and normal controls.

### Comprehensive evaluation

STRING is an online platform for searching known protein-protein interactions and integrating corresponding protein-protein interaction data. STRING was used to evaluate the PPI network of m6A regulators in breast cancer.

CCLE covers the gene expression of thousands of tumor cell lines from dozens of tissues and is a sharp tool for tumor research [14]. The corresponding CCLE data were selected and R software (version 4.1.0) was used to analyze the expression of HNRNPA2B1 in various tumor cell lines.

The Kaplan-Meier plotter is a commonly used tool for tumor survival analysis [15, 16]. To evaluate the prognostic value of HNRNPA2B1 mRNA in breast cancer. Survival outcomes included OS and DMFS. The KM plotter algorithm was used to determine the best cut-off value.

### Immunohistochemistry and result judgement

145 cases of breast cancer and 30 cases of para-cancerous normal tissues archived in the Affiliated Tumor Hospital of Xinjiang Medical University from January to December 2016. The clinical features are shown in Table 1. Related paraffin specimens were collected. All of them were female, and their average age is 45.8 ± 10.2 years. All of them were operated for BC for the first time. This experiment was approved by the Medical Ethics Committee of our hospital (K-2021054) and agreed by these 145 patients.

**Table 1:** The clinical features of patients.

**Characteristics**	**All cases**	**HNRNPA2B1**		**OR**	***p* value**
		**Low (*n* = 75)**	**High (*n* = 70)**		
Age	45.8 ± 10.2	46.3 ± 9.26	45.2 ± 11.2	0.99 (0.96; 1.02)	0.5
Menstruation					
No	104 (71.7%)	51 (68.0%)	53 (75.7%)	0.68 (0.32; 1.42)	0.397
Menopause	41 (28.3%)	24 (32.0%)	17 (24.3%)		
ER	38.2 ± 26.7	52.1 ± 22.0	23.4 ± 23.3	0.95 (0.93; 0.97)	<0.001
PR	18.8 ± 19.3	27.8 ± 19.2	9.20 ± 14.2	0.94 (0.91; 0.96)	<0.001
HER2					
No	112 (77.2%)	62 (82.7%)	50 (71.4%)	1.89 (0.86; 4.29)	0.157
Yes	33 (22.8%)	13 (17.3%)	20 (28.6%)		
Ki67	34.1 ± 18.9	23.3 ± 14.0	45.8 ± 16.4	1.10 (1.07; 1.14)	<0.001
Lymph node metastasis					
No	60 (41.4%)	48 (64.0%)	12 (17.1%)	8.38 (3.93; 19.1)	<0.001
Yes	85 (58.6%)	27 (36.0%)	58 (82.9%)		
AJCC stage					
I	27 (18.6%)	23 (30.7%)	4 (5.71%)	Ref.	<0.001
II	89 (61.4%)	45 (60.0%)	44 (62.9%)	5.40 (1.87; 20.1)	
III	29 (20.0%)	7 (9.33%)	22 (31.4%)	16.5 (4.56; 74.8)	
Histologic grade					
1	11 (7.59%)	9 (12.0%)	2 (2.86%)	Ref.	<0.001
2	94 (64.8%)	60 (80.0%)	34 (48.6%)	2.40 (0.56; 18.1)	
3	40 (27.6%)	6 (8.00%)	34 (48.6%)	22.1 (4.36; 190)	

Immunohistochemical SP method was used to detect the expression of HNRNA2B1 protein in breast cancer and benign breast adenosis. The procedure was performed strictly according to the instructions of the kit. Paraffin blocks of breast cancer and benign adenosis of the breast were cut into 4 μm thick tissue, made into white sections, dewaxed, hydrated, heat-fixed, sealed, and added with antibodies; The cells were stained with DAB kit, dehydrated, transparent, sealed, and observed under a microscope. The expression of HNRNA2B1 was mainly localized in the nucleus. IHC results were interpreted by two pathologists in a double-blind manner. The percentage of positive cells and staining intensity were observed: (1) staining intensity: no positive staining or cell chromogenic indistinguishability from the surrounding stroma was 0, light yellow was 1, yellow or brownish yellow was 2, and brown was 3 and (2) percentage of positive cells: the number of positive cells <5% as 0, 5~25% as 1, 25~75% as 2, and >75% as 3. The above two scores were multiplied as the final score of HSPA8 protein expression: 0 as negative, ≥1 as positive, 1~3 as low expression, and 4~12 as high expression.

### HNRNPA2B1 and immune response

Based on the RNAseq data of TCGA-BRCA, the correlation between HNRNPA2B1 and immune infiltration was analyzed. TIMER2.0 gene module was used to study the relationship of HNRNPA2B1 and tumor-Infiltrating Immune Cells [17]. The immune infiltration was calculated by using the ssGSEA algorithm provided in the GSVA package [18] and referring to the 24 kinds of immune cells provided by the Immunity article [19], and the analysis results were visualized with ggplot2 package. Then, we assessed the correlations between the expression of HNRNPA2B1 and immunoregulators (including immunoinhibitors, immunostimulator, and MHC molecules) by using TISIDB database (http://cis.hku.hk/TISIDB/).

### Statistical analysis

All statistical analyses were performed using R software (version 4.0.5). The code script was supplied (Supplementary File 1). The Wilcoxon’s test was applied to contrast the expression of m6A regulators between cancer and normal tissues. Lasso regression was performed by the “glmnet” package in R. The chi-square test was used to compare the relationship between m6A and Immune Response. Wilcoxon signed rank test was utilized for comparison the IHC score between tumor and the normal counterparts. And the log-rank test was employed to compare the survival probabilities between the low and high expression of target gene. The validation of the diagnostic models was assessed by receiver operating characteristic (ROC) curve. For all the analyses, a *P*-value less than 0.05 was regarded as statistically significant. The abbreviation list was shown in Supplementary Table 3.

## RESULTS

### Amplification, deletion, and mutation analysis of m6A regulators

**Figure 1:**
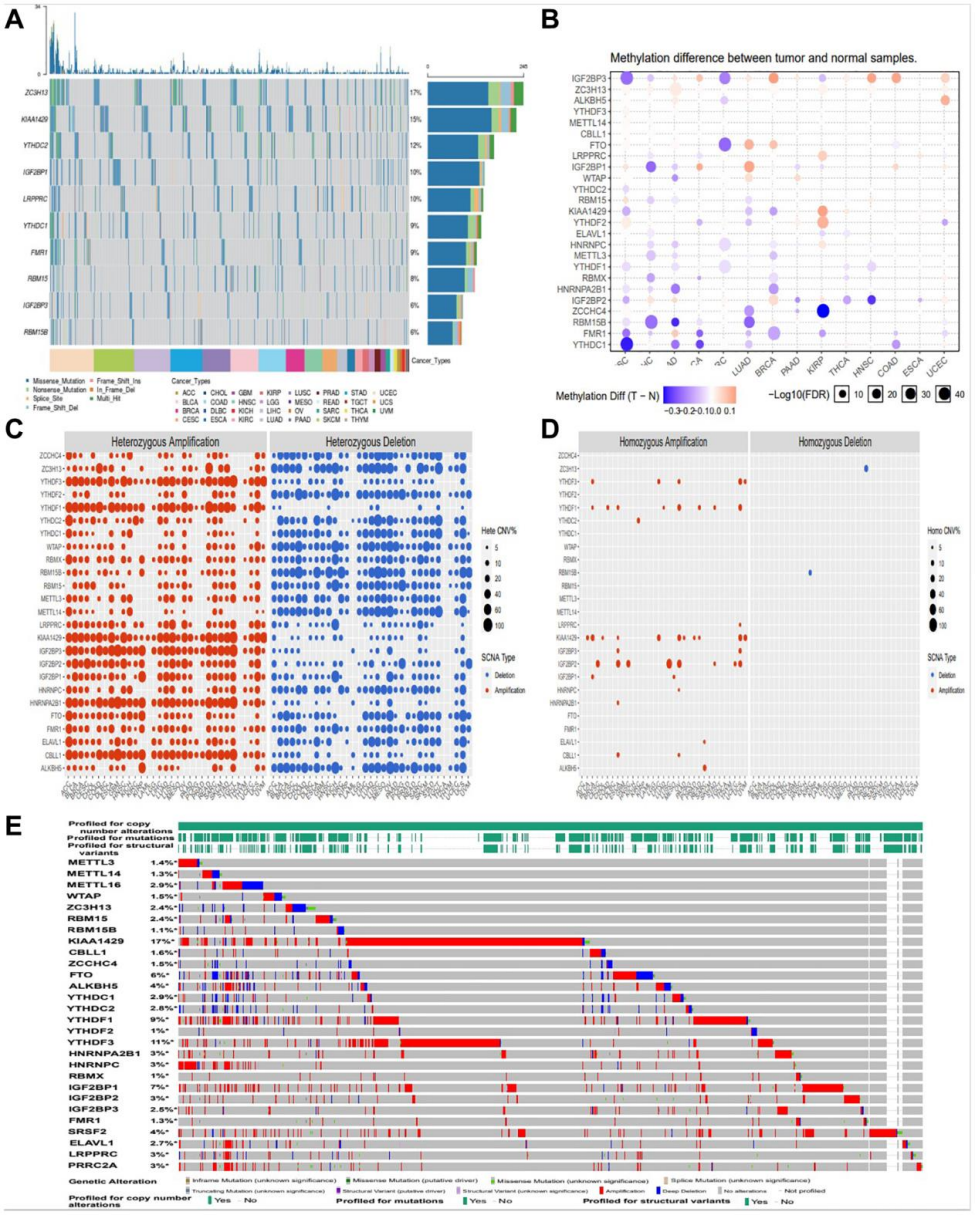
**Amplification, deletion, and mutation analysis of m6A regulators.** (**A**) The waterfall plot showed a mutation distribution of top 10 mutated genes and a SNV classification of SNV types in pan-cancer (**B**) Methylation module explores the differential methylation between tumor and paired normal, the correlation between methylation with expression and the OS affected by methylation level for selected cancer types. (**C**, **D**) Heterozygous/Homozygous CNV profile show you percentage of heterozygous/homozygous CNV including amplification and deletion percentage of heterozygous/homozygous CNV about each gene in each cancer. Only genes with >5% CNV in cancers will show corresponding points on the figure (Abbreviations: Hete Amp: heterozygous amplification; Hete Del: heterozygous deletion; Homo Amp: homozygous amplification; Homo Del: homozygous deletion; None: no CNV). (**E**) cBioPortal focus on homozygous CNV in the present.

In this study, firstly, we compared the genetic changes of top 10 m6A regulators in pan cancer (Figure 1A). We found that 28 m6A regulatory factors had different degrees of genetic change (Figure 1B). In this CNV module, we calculate the percentage of CNV, CNV correlation with mRNA of gene in each cancer type. The CNV was divided into 2 subtypes, heterozygous CNV and homozygous CNV, which represent the occurrence of CNV on only one chromosome or both two. Percentage statistic based on subtypes of CNV used GISTIC processed CNV data, and calculation of correlation used raw CNV data and mRNA RSEM data (Figure 1C, 1D). And we also found amplification, deletion and mutation of most m6A regulators in pan cancer, with KIAA1429 having the highest incidence (17%) (Figure 1E).

### The expression of M6A regulators was different in breast cancer

**Figure 2:**
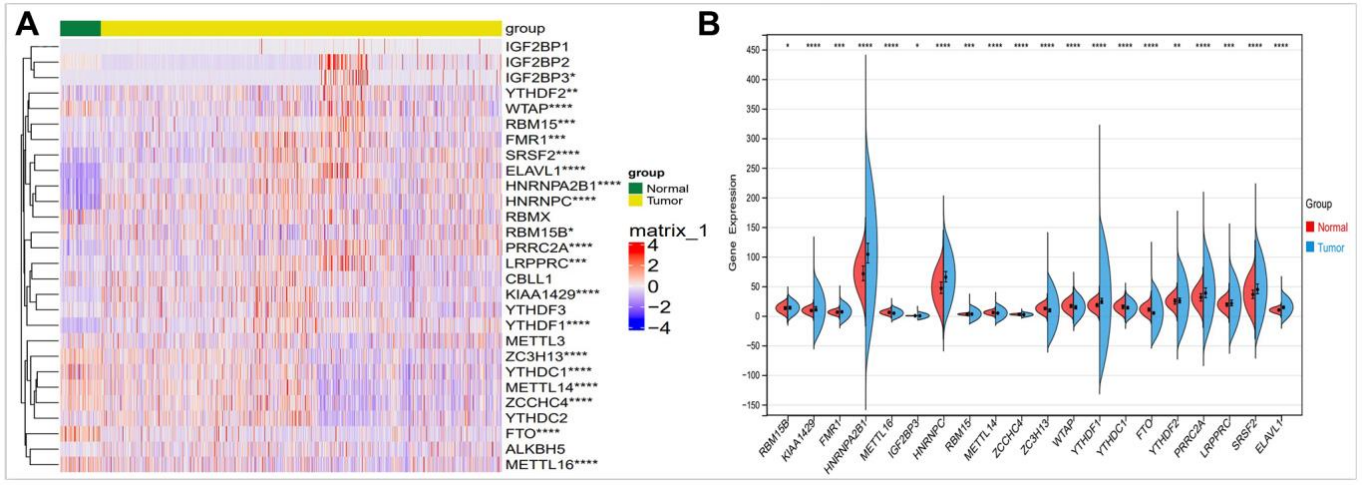
**Comparison of the expression of m6A regulators in breast cancer and normal controls.** (**A**) There were showed expression of m6A regulators in normal and tumor sample by heatmap (Red, high expression; Blue, low expression). (**B**) Vio-plot showed the significant differential expression of 20 m6A regulator genes in normal and tumor sample. The asterisks represented the statistical *p* value (^*^P < 0.05; ^**^P < 0.01; ^***^P < 0.001).

In order to evaluate the role and expression differences of m6A in breast cancer, we conducted a comparative study on the expression of 28 m6A regulators in breast cancer by using TCGA database. As shown in Figure 2A, we found differences in 20 m6A regulators expression in breast cancer. Using these differentially expressed m6A factors, we further analyzed and found that HNRNPA2B1, HNRNPC, YTHDF1, PRRCRA expression are upregulated. FTO expression is reduced in breast cancer (Figure 2B).

### Construction of diagnostic signature based on m6A regulators

**Figure 3:**
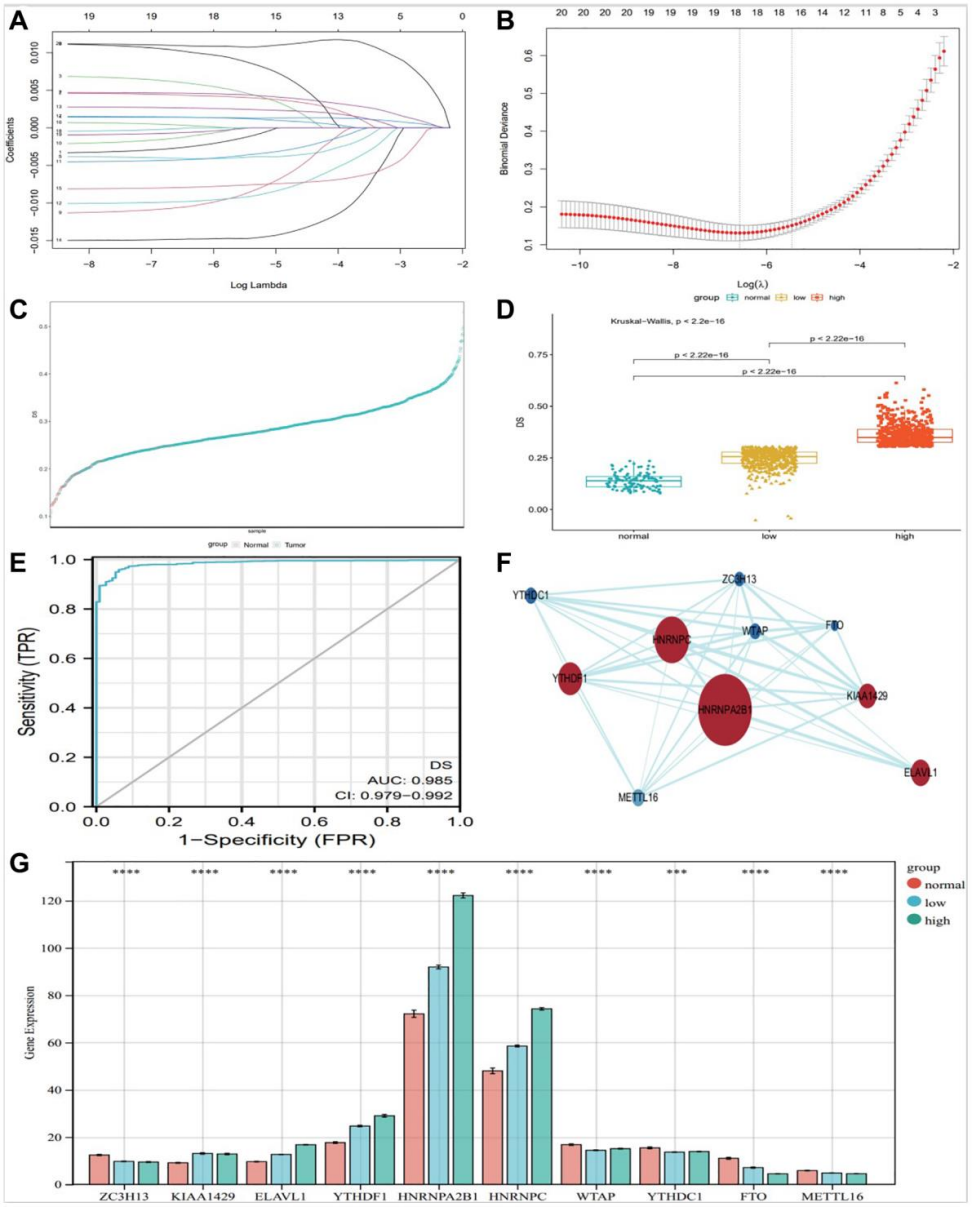
**Construction of diagnostic Signature based on m6A regulators.** The binomial deviance curve was plotted versus log (λ), where λ is the tuning parameter (**A**). LASSO coefficient profiles of clinicopathologic variables (**B**). DS distribution in normal and tumor (**C**) and between normal, low and high group (**D**). (**E**) ROC curve showed the specificity of DS diagnostic score. PPI analysis of key 10 m6A factors (**F**), and bar graph showed them expression in different groups (**G**), (^*^P < 0.05; ^**^P < 0.01; ^***^P < 0.001).

We included m6A regulators with significant differential expression into the Lasso-logistic model and analyzed their expression in TCGA-BRCA samples to screen candidate molecules with potential diagnostic value for breast cancer. The samples were randomly divided into train (Tumor 843 and normal 78) group and test (Tumor 362 and normal 35) group. The model was constructed by training group, and three potential m6A regulators were screened out in lasso regression analysis (Figure 3A, 3B). We constructed a diagnostic signature of breast cancer: DS (Diagnostic Score) = expKIAA1429 × 0.0015761555 + expHNRNPA2B1 × 0.0017949063 + expMETTL16 × −0.0015140717 + expHNRNPC × 0.0036807689 + expMETTL14 × −0.0021035106 + expZC3H13 × −0.0009608338 + expWTAP × −0.0026646221 + expYTHDF1 × 0.0018432470 + expYTHDC1 × −0.0109597582 + expFTO × −0.0070609102.

We evaluated the diagnostic predictive value of DS. The DS scores of high and low groups were significantly different from normal control group (Figure 3D). According to the DS score, Figure 3C shows the distribution of DS in normal and tumor. The DS score showed a high diagnostic predictive value (AUC = 0.978, Figure 3E).

We used STRING website (https://string-db.org/) and CytoScape software (National Resource for Network Biology, USA) to analyze the protein-protein interactions of the 10 m6A regulators (Figure 3F). Protein-protein interaction (PPI) analysis showed that HNRNPA2B1 was the key regulator. At the same time, The DS scores of the 10 key factors screened by the above method were plotted as a bar graph, showing that HNRNPA2B1 expression was most significantly different (Figure 3G).

### Pan-cancer analysis of HNRNPA2B1

**Figure 4:**
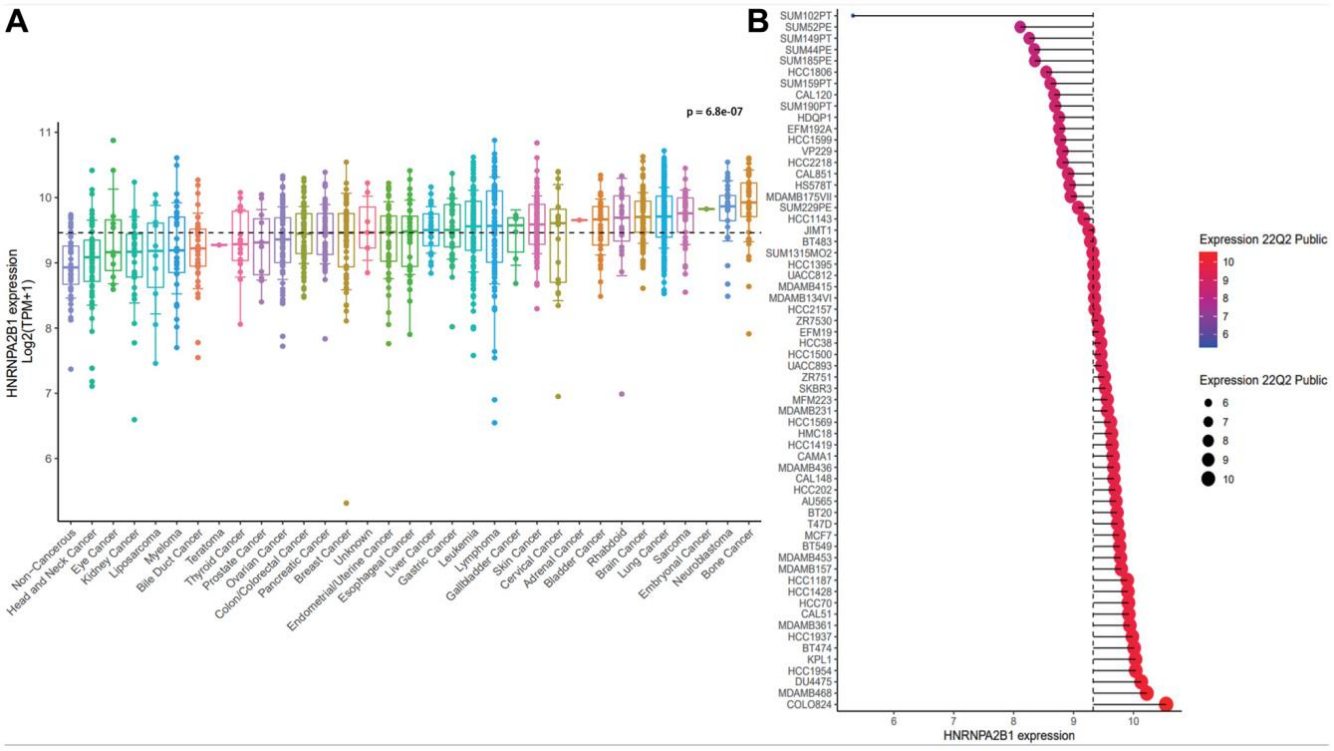
**The expression analysis of HNRNPA2B1.** (**A**) HNRNPA2B1 expression in pan-cancer cells. (**B**) HNRNPA2B1 expression in breast cancer cells. *p* value was calculated Kruskal-Wallis H Test.

By analyzing the expression of HNRNPA2B1 in the majority of tumor cells in the CCLE database Supplementary Table 4, it was confirmed that HNRNPA2B1 expression was significantly higher in breast cancer (Figure 4A). HNRNPA2B1 was expressed at higher levels in breast cancer cell lines (e.g., COLO824, MDAMB468, DU4475) than in other BC molecular subtypes (e.g. HCC2218, SUM185PE) (Figure 4B).

### Immunohistochemical (IHC) expression of HNRNPA2B1

**Figure 5:**
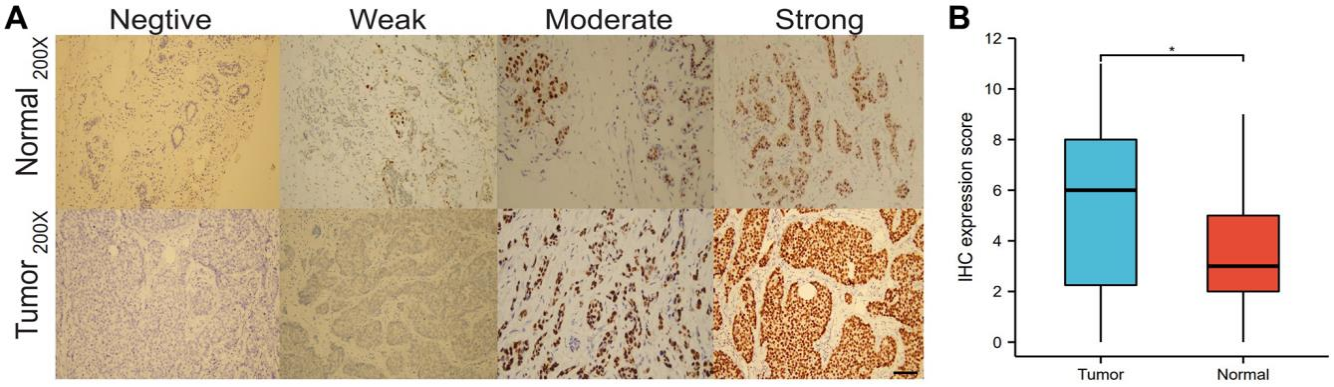
**IHC expression of HNRNPA2B1.** IHC expression of HNRNPA2B1 in normal and tumor tissues (**A**), and box-plot showed IHC expression score between tumor and normal (**B**). (^*^P < 0.05; ^**^P < 0.01; ^***^P < 0.001).

We analyzed the differences in HNRNPA2B1 expression between the immunohistochemical results of 30 normal and 40 breast cancers. HNRNPA2B1 was mainly found in the nucleus and partially in the cytoplasm. The positive criterion was obvious brown particles found under the microscope (Figure 5A). HNRNPA2B1 protein was highly expressed in cancer tissues compared with normal tissues (Figure 5B).

### Correlation analysis HNRNPA2B and clinical parameters

**Figure 6:**
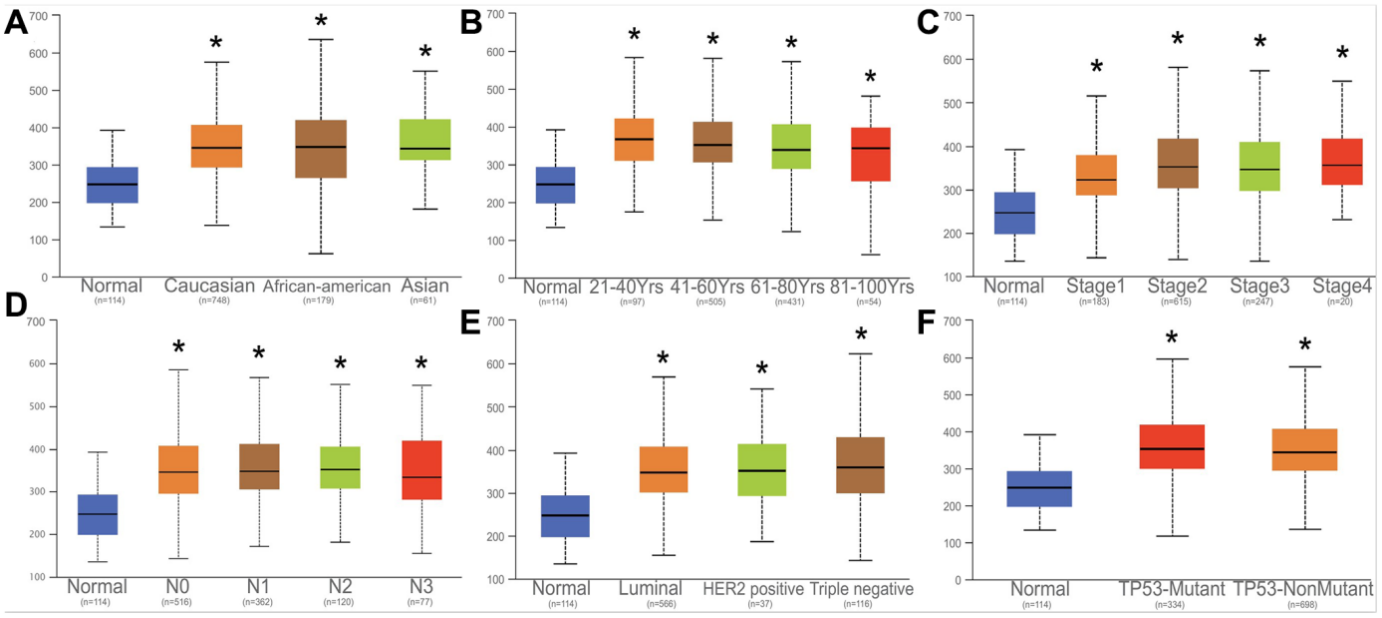
**Correlation analysis with HNRNPA2B1 and clinical parameters.** Correlation analysed with HNRNPA2B1 expression and different counties (**A**), age (**B**), tumor stage (**C**), N stage (**D**), Breast cancer subtype (**E**), TP53-mutation (**F**).

The expression of HNRNPA2B1 among Caucasian, Asian, and African American races, clinical stage, lymph node stage, and tumor subtypes, TP53-mutation were further analyzed. Notably, HNRNPA2B1 expression was increased to varying degrees in various clinical data of breast cancer patients (Figure 6A–6F).

### HNRNPA2B1 and prognosis analysis

**Figure 7:**
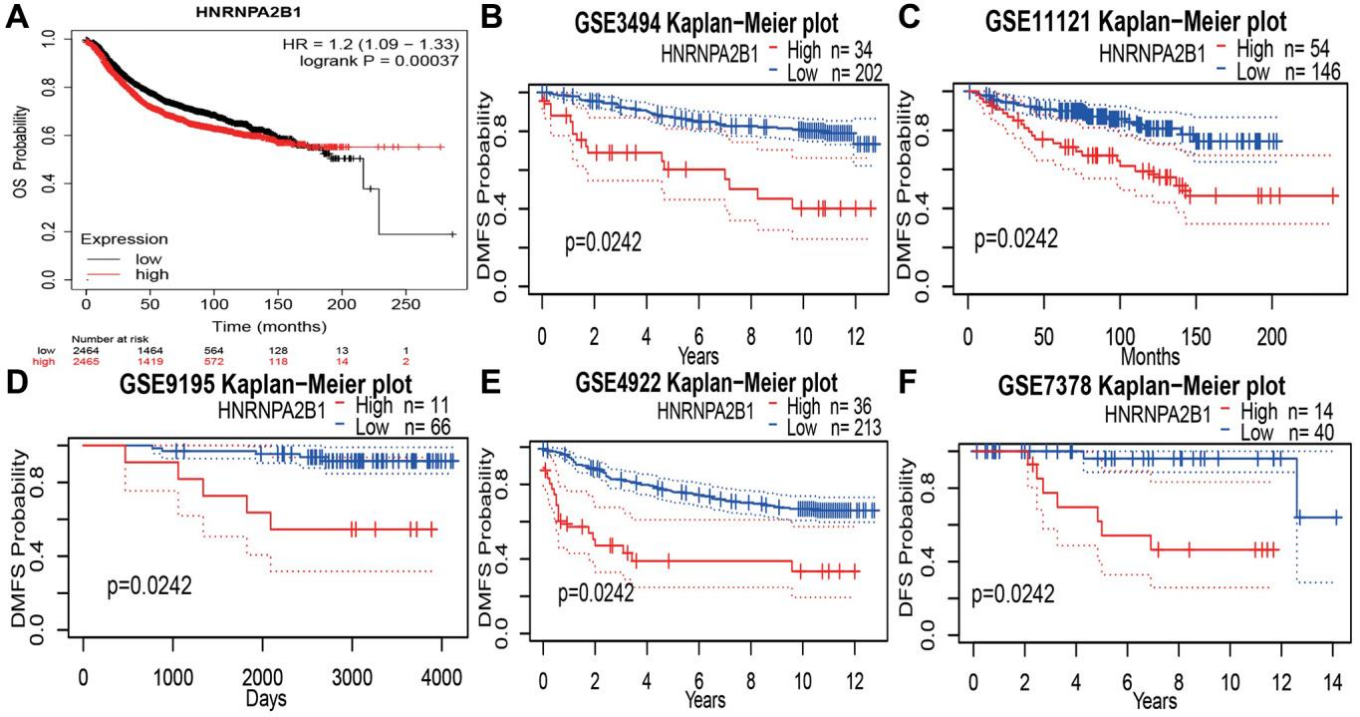
**Prognosis analysis with HNRNPA2B1 expression.** (**A**) OS analysis of HNRNPA2B1 in BC patients by Kaplan-Meier Plotter online website. DMFS analysis of HNRNPA2B1 in GSE3494 (**B**), GSE11121 (**C**), GSE9195 (**D**), GSE4922 (**E**), and DFS analysis in GSE7378 (**F**).

Online database, showing a poor prognosis with high HNRNPA2B1 expression. Kaplan-Meier Plotter online website, OS results, showed that 2465 cases of high expression/2464 cases of low expression, the prognosis of high expression was poor, and the difference was statistically significant Figure 7A. Compared with the low expression group, the high expression group increased the risk of death by 1.2 times. At the same time, 5 data sets of GEO database were found, DMFS: Distant metastasis-free survival rates all showed poor prognosis with high expression Figure 7B–7F.

### Cox analysis of HNRNPA2B1 expression

**Figure 8:**
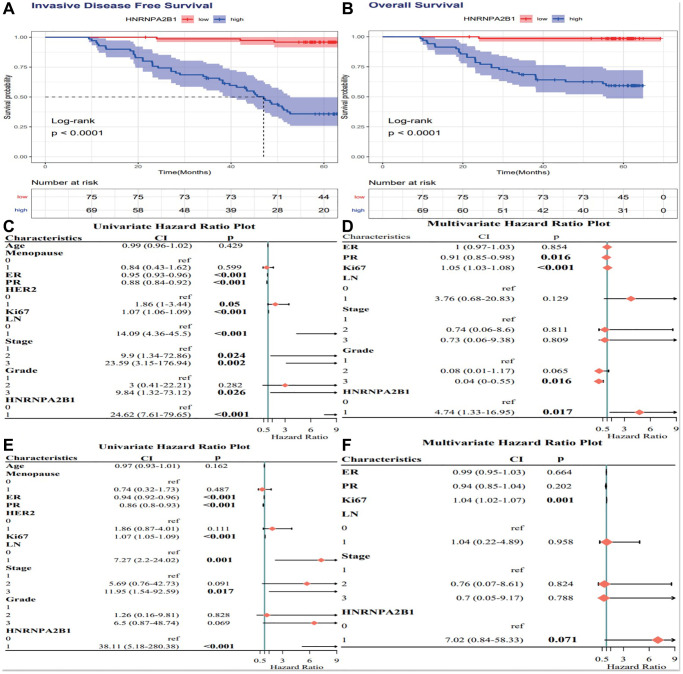
**Cox analysis of HNRNPA2B1 expression.** IDFS analysis (**A**) and OS analysis (**B**) with HNRNPA2B1 expression. Cox regression analysis for IDFS (**C**, **D**) and OS (**E**, **F**).

High HNRNPA2B1 expression is associated with poor prognosis of breast cancer, *P* < 0.001 (Figure 8A, 8B). By Cox analysis, HNRNPA2B1 was a risk factor for the prognosis of breast cancer (Figure 8C–8F).

### HNRNPA2B1 and immune

**Figure 9:**
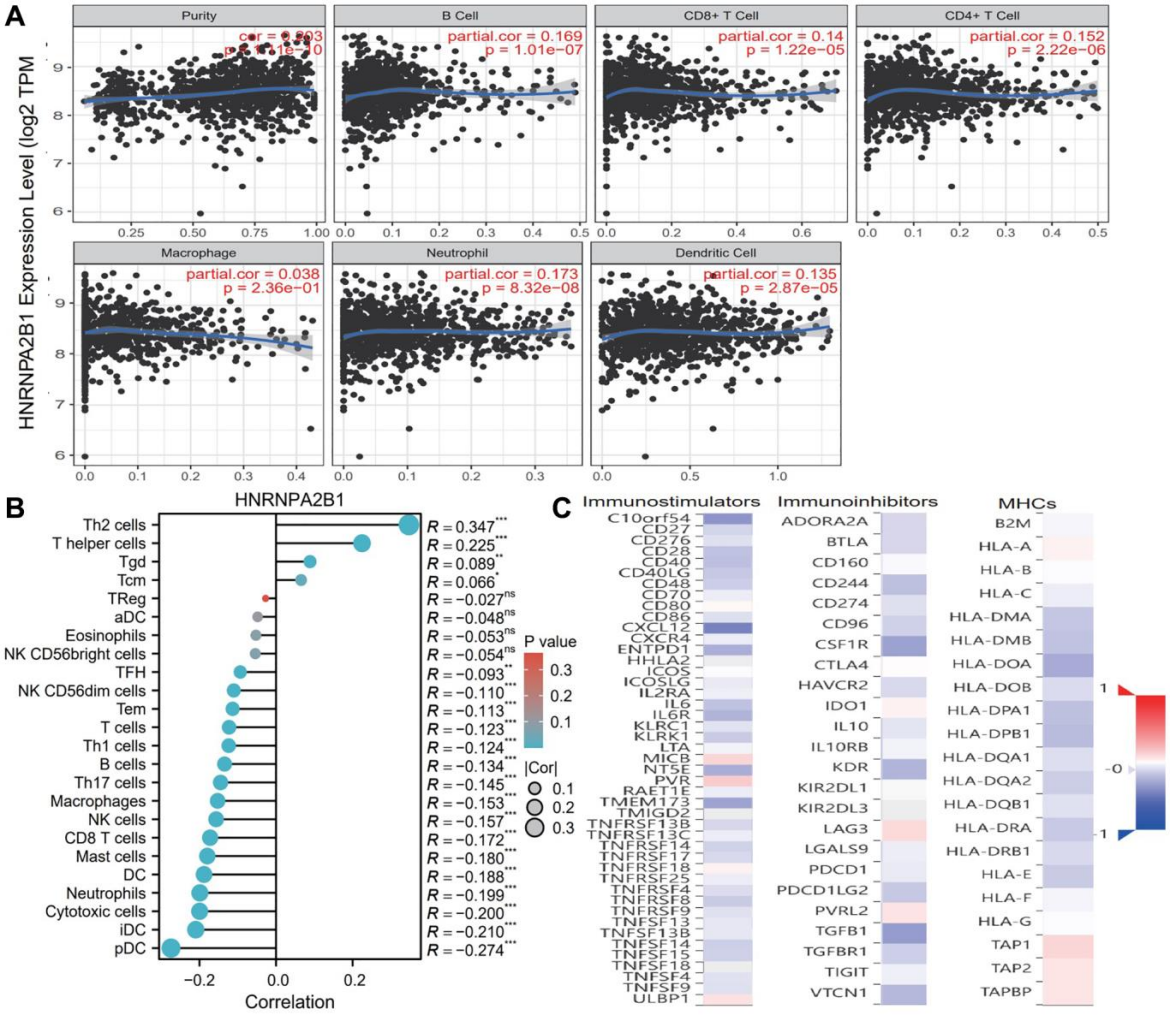
**Immune infiltration analysis with HNRNPA2BP1.** (**A**) Examine the correlation between HNRNPA2BP1 and the level of immune cell infiltration in breast cancer based on TIMER database. (**B**) The correlation analysis with 24 immune cells and HNRNPA2BP1 expression (^*^P < 0.05; ^**^P < 0.01; ^***^P < 0.001). (**C**) The correlation analysis with immunostimulators, immunoinhibitors and MHCs (Red, positive correlation; Blue, negative correlation).

Based on TIMER database, we examined the correlation between m6A regulators and the level of immune cell infiltration in breast cancer. HNRNPA2B1 (Figure 9A) was associated with purity (cor = 0.203, *p* = 1.11e-10), B cell (cor = 0.169, *p* = 1.01e-07), CD8+ T cell (cor = 0.14, *p* = 1.22e-05), CD4+ T cell (cor = 0.152, *p* = 2.22e-06), Neutrophill (cor = 0.173, *p* = 8.32e-08), Dendritic Cell (cor = 0.135, *p* = 2.87e-05). As shown in Figure 9B, HNRNPA2BP1 was positively correlated with Th2 and T helper cells, and negatively correlated with pDC, iDC, T cell and B cell.

To further explore the effects of m6A regulators on tumor immune response, we calculated the correlation between the expression of m6A regulators and immune regulators. As shown in Figure 9C, the expression level of HNRNPA2B1 was negatively correlated with immunoinhibitors, immunostimulators, and MHC molecules expressions in breast cancer.

## DISCUSSION

Breast cancer is heterogeneous, and genetic or epigenetic factors play an indispensable role in its occurrence and development [20]. At present, the early diagnosis and precise individualized treatment of BRC are still the biggest challenges. Therefore, the identification of consistently altered genomic signatures is critical in BRC basic and clinical research. To discover novel therapeutic targets, we investigated the expression patterns of m6A-associated genomic targets in BRC at the mRNA and protein levels.

N6-Methyladenosine (m6A) RNA modification as a reversible epigenetic modification, is the most common form in post-transcriptional regulation [21]. It’s in messenger RNAs as well as in non-coding RNAs. m6A methylation regulatory protein plays crucial part in nearly all vital bioprocesses [22]. The dysregulation of m6A is involved in the occurrence and progression of cancer [23]. A great number of studies revealed that dysregulated m6A methylation modulators were closely related to the occurrence and development of tumors in different types of cancer [9, 24, 25]. m6A methylation regulatory proteins are important regulatory factors and play a key part in tumorigenesis and development [26], its expression level often directly determines the pathological process of the tumor [27]. So it provides more possibilities for early diagnosis and treatment of cancer [13, 14]. Previous studies on the correlation between m6A methylation and breast cancer are mostly limited to individual molecules [10]. HNRNPA2B1 by recognition and binding Specific RNA substrates and DNA motifs are involved in RNA transcription, splicing, stability, and translation, and regulate the expression of a variety of genes [28]. Elevated HNRNPA2B1 levels in tumors accelerate pre-mRNA processing through RNA binding, suggesting a critical role for HNRNPA2B1 in cancer development. HNRNPA2B1 is highly expressed in multiple types of tumor tissues. Heterogeneous ribonucleoprotein (HNRNP) A2B1 has two isoforms, A2 and B1, which are the products of alternative splicing of the precursor mRNA of the same gene. As an RNA-binding protein, HNRNPA2B1 is involved in carcinogenesis through its interaction with other proteins. HNRNPA2B1 is upregulated in multiple tumors and affects their biological processes, and is involved in various cellular processes such as cancer cell metabolism, migration, invasion, proliferation, survival and apoptosis by RNA processing, splicing, trafficking, and the stability of many downstream target genes. HNRNPA2B1 is highly expressed in many cancers, such as pancreatic, breast and prostate cancers, and malignant gliomas, where HNRNPA2B1 plays an important role in carcinogenesis, invasion and metastasis.

In recent years, several studies have revealed the role of m6A regulators in breast cancer. Studies have shown that m6A “eraser” FTO is significantly up-regulated in breast cancer, which can promote breast cancer cell proliferation, colony formation and reduce apoptosis [29]. We systematically analyzed the expression of 28 m6A regulators in breast carcinoma, established a diagnostic prediction model by lasso regression analysis, and well validate with GEO datasets to predict the risk of breast cancer.

We found that there were essential differences in the expression of the m6A regulators between breast cancer and normal controls. The relationship between m6A regulators and Breast Cancer have been reported in studies [20, 27, 29]. It was found that the abnormal expression of HNRNPA2B1 was considerably related to the occurrence of breast cancer by lasso regression analysis. The diagnostic model has been constructed with the three m6A regulators, and the diagnostic value of Breast Cancer was well validated In TCGA (AUC = 0.964), GEO datasets also verified the potential signature of HNRNPA2B1 in the diagnosis of Breast Cancer. m6A dynamically regulates the modification level through the activities of methyltransferase and demethylase, and recruits RNA-binding protein to complete biological functions. The m6A reader HNRNPA2B1 directly bind to m6A modification site and regulate alternative splicing and primiRNA processing [30]. HNRNPA2B1 promotes the progression of Esophageal Cancer by up-regulating ACLY and ACC1 [31], contributes to epithelial-to-mesenchymal transition by MST1R-Akt axis in head and neck cancer [32], promotes apoptosis by regulating Lin28B in ovarian cancer [33], In breast cancer, HNRNPA2B1 is regulated by MIR-204 and affects the invasion and metastasis of breast cancer cells [34].

The protection of harmful pathogens depends on the activation of the immune system, which relies on the strict regulation of gene expression. Recently, RNA-modified N6-methyladenosine (m6A) has been found to play a vital role in this regulation. m6A controls various aspects of immunity, including immune recognition, activation of innate and adaptive immune responses, and determination of cell fate [35]. FTO plays critical roles in cancer stem cell maintenance and immune evasion [36]. In this study, it was found that HNRNPA2B1 was significantly correlated with the level of immune cell infiltration in breast cancer, as well as with the Stromal Score, ESTIMATE Score and immune Score. Through TISIDB database, we found that the three m6A regulators had closely connection with immunoinhibitors, immunostimulators and MHC molecules in breast cancer. It is also suggested that the occurrence of breast cancer is related to the immune disorder caused by the abnormal expression of m6A.

## CONCLUSION

Systematic analysis of 28 m6a regulators identified 10 key genes and constructed a diagnostic score. We found that HNRNPA2B1 was significantly differentially expressed in breast cancer and correlated with breast cancer prognosis and immune infiltration.

## Supplementary Materials

Supplementary File 1

Supplementary Table 1

Supplementary Tables 2 and 3

Supplementary Table 4
