# Supplementary material for: HNRNPA2B1 is a potential biomarker of breast cancer related to prognosis and immune infiltration
Source: Aging (Albany NY). 2023 Sep 5;15(17):8712–28. doi: 10.18632/aging.204992 (PMC10522385; doi:10.18632/aging.204992)
Supplement: Supplementary File 1 [file aging-15-204992-s001.pdf]

SUPPLEMENTARY MATERIALS

Supplementary File 1.

```
### 读入数据
data<-read.table(file="TCGA_BRCA_1222samples_FPKM.txt",header = TRUE,sep = "\t")

####   指定 m6A 相关基因
m6A<-
c("METTL3","METTL14","METTL16","WTAP","ZC3H13","RBM15","RBM15B","KIAA1429","CBLL1","ZCCHC4"
,"FTO","ALKBH5","YTHDC1","YTHDC2","YTHDF1","YTHDF2","YTHDF3","HNRNPA2B1","HNRNPC","RBMX"
,"IGF2BP1","IGF2BP2","IGF2BP3","FMR1","SRSF2","ELAVL1","LRPPRC","PRRC2A","ELF3","SampleType","Vita
l","Followup")

### 匹配 M6A
data<-data[data$GeneName%in%m6A,]
head(data[,1:10])
data<-data[,-c(2,3,4)]
rownames(data)<-data$GeneName
data<-data[,-1]

###  分组
group<-data[1,]
group<-group[,-c(1)]
group<-as.data.frame(t(group))
group$group<-ifelse(group$SampleType=="Solid Tissue Normal","Normal","Tumor")
group$sample<-rownames(group)
group<-group[,-c(1)]
sum(group$group=="Normal")

####   差异分析
library(limma)
dim(group)
BRCA_T_P_des<-as.factor(group$group)#把分组 charater 转化为 factor

## 构建的 design 的模型
design1<-model.matrix(~0+factor(BRCA_T_P_des),levels=levels(BRCA_T_P_des))
colnames(design1)<-c("P", "T")
rownames(design1) <- group$sample

setequal(colnames(data),rownames(design1))
sapply(data,list)
class(data[,1222])
dim(data)
for (i in 1:1222) {data[,i]<-as.numeric(data[,i])}

fit_sub1 <- lmFit(data, design1)
cont.matrix_sub1 <- makeContrasts(T-P, levels=design1)#Designing Contrast Matrix for group Differentiation
fit2_sub1 <- contrasts.fit(fit_sub1, cont.matrix_sub1)
fit3_sub1 <- eBayes(fit2_sub1)
```

```
allGenes_sub1 <- topTable(fit3_sub1,number=Inf)
dim(allGenes_sub1)
colnames(allGenes_sub1)

diff_m6A<-subset(allGenes_sub1,adj.P.Val <0.05)
write.table(allGenes_sub1,file="m6A_diff.txt",sep="\t") #保存
```
