# Supplementary material for: HNRNPA2B1 is a potential biomarker of breast cancer related to prognosis and immune infiltration
Source: Aging (Albany NY). 2023 Sep 5;15(17):8712–28. doi: 10.18632/aging.204992 (PMC10522385; doi:10.18632/aging.204992)
Supplement: Supplementary Tables 2 and 3 [file aging-15-204992-s003.pdf]

Supplementary Tables

Supplementary Table 2. The differentially expressed genes of 28 m6A regulators in breast cancers.

	logFC	AveExpr	*t*	*P*.Value	adj.*P*.Val	B
ELAVL1	−2253.9	5999.799	−10.3422	4.42E-24	1.24E-22	−4.53448
HNRNPA2B1	−16651.9	46840.95	−9.72001	1.49E-21	2.08E-20	−4.54111
HNRNPC	−8674.39	28268.85	−9.11379	3.21E-19	3.00E-18	−4.54729
FTO	3697.178	4789.598	8.87077	2.56E-18	1.79E-17	−4.54968
YTHDF1	−1933.6	5154.266	−6.98663	4.64E-12	2.60E-11	−4.56649
PRRC2A	−5579.43	21267.07	−6.37175	2.65E-10	1.24E-09	−4.57126
SRSF2	−1947.59	8291.673	−5.90573	4.56E-09	1.82E-08	−4.57461
KIAA1429	−2750.08	8268.055	−4.62723	4.10E-06	1.44E-05	−4.58263
METTL16	527.5715	2539.268	4.530494	6.47E-06	2.01E-05	−4.58316
ZC3H13	1515.896	6110.634	4.221042	2.61E-05	7.32E-05	−4.5848
METTL14	327.0938	1920.021	3.724829	0.000204	0.00052	−4.58719
RBM15	−137.619	855.7333	−3.54791	0.000403	0.000941	−4.58798
ZCCHC4	91.72787	560.0438	3.519403	0.000449	0.000966	−4.5881
LRPPRC	−2388.43	13240.86	−3.32246	0.000919	0.001777	−4.58892
FMR1	−646.323	3732.624	−3.31247	0.000952	0.001777	−4.58896
YTHDF2	−688.938	6016.962	−2.81331	0.004983	0.00872	−4.59084
WTAP	595.766	5206.888	2.667432	0.007745	0.012757	−4.59133
RBM15B	−484.218	5392.743	−2.63439	0.008536	0.013279	−4.59144
IGF2BP3	−58.6925	63.97688	−2.31847	0.02059	0.030343	−4.59241
YTHDC1	515.9573	5896.497	2.253596	0.0244	0.03416	−4.59259
YTHDF3	−696.151	6453.121	−1.91027	0.056334	0.075112	−4.59348
CBLL1	−229.844	2518.998	−1.81126	0.070348	0.089534	−4.5937
IGF2BP2	156.2446	461.379	1.567333	0.117298	0.142798	−4.59421
YTHDC2	−242.96	2933.263	−1.47107	0.141532	0.16512	−4.5944
IGF2BP1	−47.6794	47.29232	−1.33748	0.181316	0.203074	−4.59463
ALKBH5	−280.059	6332.652	−1.07442	0.282847	0.304605	−4.59502
RBMX	−269.398	11703.21	−0.63531	0.525346	0.544803	−4.59549
METTL3	−3.84894	2251.994	−0.0375	0.970094	0.970094	−4.59574

Supplementary Table 3. The abbreviation list.

m6A	N6-methyladenosine
HNRNPA2B1	Heterogeneous nuclear ribonucleoprotein A2/B1
TCGA	The Cancer Genome Atlas (https://cancergenome.nih.gov/)
BRCA	Breast Cancer
GEO	Gene Expression Omnibus
DEG	Differentially expressed gene
KM	Kaplan-Meier plotter (https://kmplot.com/analysis/)
ROC	Receiver operating characteristic
CNV	Copy number variations
IHC	Immunohistochemical
OS	Overall survival
DMFS	Distant metastasis-free survival rates
